# Revolutionizing Breast Cancer Detection With Artificial Intelligence (AI) in Radiology and Radiation Oncology: A Systematic Review

**DOI:** 10.7759/cureus.57619

**Published:** 2024-04-04

**Authors:** Zubir S Rentiya, Shobha Mandal, Pugazhendi Inban, Hemika Vempalli, Rishika Dabbara, Sofia Ali, Kirpa Kaur, Abiodun Adegbite, Tarsha A Intsiful, Malavika Jayan, Victor A Odoma, Aadil Khan

**Affiliations:** 1 Radiation Oncology & Radiology, University of Virginia School of Medicine, Charlottesville, USA; 2 Neurology, Regional Neurological Associates, New York, USA; 3 Internal Medicine, Salem Internal Medicine, Primary Care (PC), Pennsville, USA; 4 General Medicine, Government Medical College, Chennai, IND; 5 Internal Medicine, Narayana Medical College, Nellore, IND; 6 Internal Medicine, Kamineni Institute of Medical Sciences, Hyderabad, IND; 7 Medicine, Peninsula Medical School, Plymouth, GBR; 8 Medicine, Howard Community College, Ellicott City, USA; 9 Medicine and Surgery, University of Ibadan, Oyo, NGA; 10 Radiology, College of Medicine, University of Ghana Medical Center, Accra, GHA; 11 Internal Medicine, Bangalore Medical College and Research Institute, Bangalore, IND; 12 Research, California Institute of Behavioral Neurosciences & Psychology, Fairfield, USA; 13 Cardiovascular Medicine/Oncology (Acuity Adaptable Unit), Indiana University Health, Bloomington, USA; 14 Trauma Surgery, Order of St. Francis (OSF) St Francis Medical Centre, University of Illinois Chicago, Peoria, USA; 15 Cardiology, University of Illinois at Chicago, Chicago, USA; 16 Internal Medicine, Lala Lajpat Rai (LLR) Hospital, Kanpur, IND

**Keywords:** digital breast tomosynthesis, computer-aided detections, artificial intelligence (ai), mammography, 3d tomosynthesis

## Abstract

The number one cause of cancer in women worldwide is breast cancer. Over the last three decades, the use of traditional screen-film mammography has increased, but in recent years, digital mammography and 3D tomosynthesis have become standard procedures for breast cancer screening. With the advancement of technology, the interpretation of images using automated algorithms has become a subject of interest. Initially, computer-aided detection (CAD) was introduced; however, it did not show any long-term benefit in clinical practice. With recent advances in artificial intelligence (AI) methods, these technologies are showing promising potential for more accurate and efficient automated breast cancer detection and treatment. While AI promises widespread integration in breast cancer detection and treatment, challenges such as data quality, regulatory, ethical implications, and algorithm validation are crucial. Addressing these is essential for fully realizing AI's potential in enhancing early diagnosis and improving patient outcomes in breast cancer management. In this review article, we aim to provide an overview of the latest developments and applications of AI in breast cancer screening and treatment. While the existing literature primarily consists of retrospective studies, ongoing and future prospective research is poised to offer deeper insights. Artificial intelligence is on the verge of widespread integration into breast cancer detection and treatment, holding the potential to enhance early diagnosis and improve patient outcomes.

## Introduction and background

Breast cancer detection and diagnosis

Breast cancer stands as a significant global health issue, ranking as the most prevalent malignancy among women worldwide and claiming the second spot of death in the United States [1,2]. Approximately half a million women die every year due to breast cancer. In 2020, there were 2.3 million women diagnosed with breast cancer and 685,000 deaths globally [3]. Over the last three decades, multiple randomized clinical trials have supported the idea of breast cancer screening being done with mammography [4,5]. The prevalence of breast cancer in the screening population is <1%. Screening mammography has a sensitivity of over 85% and a specificity of 90% [6]. Despite advancements that have led to a 20%-40% reduction in mortality rates, breast cancer remains the foremost cause of female cancer-related deaths worldwide.

Various countries have adopted different approaches to breast cancer screening using mammography. In the United States, women can either initiate the screening process themselves or be referred by their healthcare providers to specialized breast screening centers. These centers, often institution-based, have developed their unique protocols and regulations for interpreting mammography images. Some centers provide immediate image readings while the patient waits, while others perform readings a day or two after image acquisition. If a suspicious lesion is identified during these readings, further investigations are initiated.

In contrast, many European screening centers employ a distinctive practice where cases are independently assessed by two radiologists or graders. In cases where these two professionals disagree on their interpretations, they engage in discussions to reach a consensus. Alternatively, they may consult a senior radiologist or reader to provide the final interpretation, which is binding. This collaborative approach has demonstrated improvements in the detection of breast cancer [7, 8]. 

Current screening methods' existing challenges and restrictions emphasize the immediate requirement for creative solutions. This necessity is where artificial intelligence (AI) emerges as a significant factor, potentially transforming breast cancer detection and diagnosis. Artificial intelligence, specifically utilizing deep learning and convolutional neural networks, has demonstrated the potential to improve the precision and speed of breast cancer screening. Its ability to analyze extensive and intricate datasets surpasses human abilities, potentially recognizing subtle patterns that suggest early-stage cancer and might otherwise go unnoticed by human observers. However, the integration of AI into clinical practice is not without challenges. One of the primary concerns is the need for standardized reporting algorithms for AI-generated information [9].

The current AI landscape in radiology features many systems and approaches, each with its unique methodology and output. While a testament to the field's innovation, this diversity poses significant challenges in clinical integration. There is a critical need for standardization in how AI systems process data and report findings to ensure consistency, reliability, and interpretability across different platforms and clinical settings. Furthermore, the involvement of AI in the identification of breast cancer goes beyond the mere examination of images. It also encompasses the management of patients, as AI can assist in forecasting the development of the illness and tailoring individualized care strategies. Hence, integrating AI into the diagnosis and treatment of breast cancer requires a comprehensive approach that considers not only advancements in technology but also ethical, legal, and practical considerations in the healthcare field.

## Review

Methodology

This systematic review adhered to the Preferred Reporting Items for Systematic Reviews and Meta-Analyses (PRISMA) statement and followed standard practices in the field. A comprehensive search was conducted across the Medical Literature Analysis and Retrieval System Online (MEDLINE) bibliographic database, PubMed, Google Scholar, Cumulative Index to Nursing and Allied Health Literature (CINAHL), and Scopus from January 1, 2000, to December 1, 2023. Two researchers independently conducted the literature search and assessed the eligibility of the identified papers based on their titles and abstracts. Subsequently, the full texts of potentially relevant publications were retrieved and evaluated for inclusion. Any disagreements among the researchers were resolved through discussion and consensus. Following this, two independent reviewers extracted and verified the data. A narrative synthesis approach was employed to analyze the findings, involving the summarization and presentation of the included research results in a logical and comprehensible manner.

**Figure 1:**
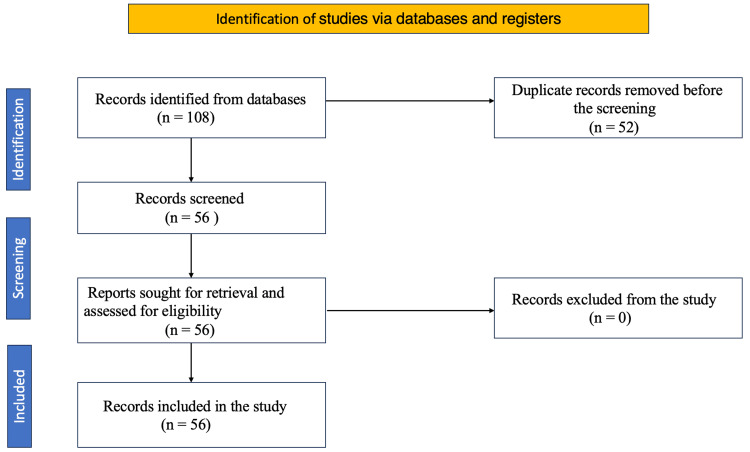
A PRISMA flow diagram outlining the study selection process PRISMA: Preferred Reporting Items for Systematic Reviews and Meta-Analyses

The initial search yielded 108 potentially relevant studies. After removing duplicates and screening titles, abstracts, and full texts, the eligibility of 56 full-text publications was assessed, as depicted in Figure 1.

Modalities of breast imaging

Digital Mammography

Mammography involves capturing a single, two-dimensional image of the breast. Before the 21st century, breast cancer screening images were obtained using film-screen mammography. However, the advent of digital mammography in clinical practice replaced film with a digital X-ray detector. This transition allowed radiologists the convenience of adjusting window leveling and magnification digitally, eliminating the need for magnifying glasses and significantly enhancing cancer detection accuracy. Moreover, digital mammography has demonstrated superior performance in breast cancer screening among specific demographic groups [10,11].

The image acquisition protocol for digital mammography typically includes obtaining two views of each breast: the cranio-caudal and the medio-lateral oblique views. This standardized imaging protocol helps minimize the chances of normal tissue overlapping malignant tissue, thus enabling radiologists to effectively detect lesions. Essentially, radiologists review both views simultaneously to ascertain whether a potential lesion identified in one view correlates with findings in the other view or if it can be dismissed as a random tissue superposition.

While reading digital mammograms for breast cancer screening purposes, the radiologist reports calcifications, mass, asymmetry, focal asymmetry, architectural distortion, and associated features [11]. In clinical practice, one of the most important biomarkers while interpreting mammograms is the detection of any change from a previous image. Therefore, during readings, the comparison of current images to prior studies is very critical. This improves sensitivity and specificity [12-16]. However, there have been a few studies where the review of prior images has shown no significant change in the outcome of breast cancer screening [12-16]. Thus, in our opinion, any deep learning algorithm for breast cancer screening should be able to assist a radiologist in determining a suspicious lesion, whether prior images are present or not.

Digital Breast Tomosynthesis (DBT)

The introduction of DBT came about in February 2011 [17]. Digital breast tomosynthesis is a pseudo-3D imaging tool that allows the radiologist to navigate the breast tissue in thin slice reconstructed images and provides significant gains in the detection of masses and architectural distortion, which a 2D digital mammography may not be able to show [18]. Hence, DBT has the potential to improve both sensitivity and specificity of breast cancer screening by reducing patient anxiety, money, and time of being called back compared to using digital mammography, which has moderate sensitivity (67.3% to 93.3%) [19-25].

Although using both imaging algorithms would be advantageous in increasing the sensitivity of breast cancer detection and reducing the number of false positives, it would also increase the dose of radiation the breast receives. Skaane et al. performed a digital mammography plus DBT protocol in their study; the radiation dose level for the combined examination was set below limits approved by the U.S. Food and Drug Administration (FDA) [21], thus constituting an adequate risk level for patient safety. 

More importantly, one major drawback from a reader’s perspective is the increase in interpretation time it takes compared to digital mammography alone or combined. It has been reported multiple times that DBT takes twice the time required for reading a digital mammogram. Skaane et al. demonstrated the mean interpretation time as 45 seconds for mammography alone and 91 seconds for mammography plus DBT [21]. Additionally, two retrospective studies by Good et al. and Gur et al. showed the time to interpret examinations using DBT alone or full-field digital mammography plus DBT combined was longer than when interpreting mammography [26, 27]. However, many breast screening centers have incorporated the use of DBT into routine screening protocols, even if optimization of interpretation efficiency is still needed.

Recently, some AI tools have been developed to reduce reading time while maintaining reader performance [28, 29]. The tools try to ease lesion detection throughout the DBT slice stack, so the radiologist will only look at those slices that the AI points out. 

The rise of deep learning convolutional neural networks

Over the years, computer-aided detection (CAD) systems have been used. Initially, CAD showed promising results; however, over time, it was shown that it has adversely affected radiologists’ performance, increasing the rate of recalls without improving the detection rate of breast cancers. Several studies have reported that CAD does not improve the diagnostic accuracy of mammography [30, 31].

Convolutional neural networks are a special type of machine learning architecture that has characteristic layers set up in a kernel. Compared to classic CAD, in convolutional neural networks, the determination of what image features are indicative of a lesion being present is achieved by the algorithm itself during its training process. Whereas in CAD, it is the input of the programmer. Thus, the program is not taught what breast cancer looks like, but it teaches itself what it looks like. The training is done by providing the model with many examples of images with and without cancer present. As the images are fed, the deep learning network adjusts the internal parameters to minimize the difference between the calculated outputs and the actual image.

Artificial Intelligence Algorithms Used in Breast Cancer Detection

Convolutional algorithms for breast cancer screening need to search for both soft tissue lesions and calcifications. The algorithm for breast cancer screening has to be trained differently than regular convolution algorithms. Multiple studies were done to compare them with traditional CAD, showing an increase in sensitivity of 83.2%-89.3% and 85.2%-93.0% for suspicious and malignant lesions, respectively [32].

Initially, at the time of introduction, the convolutional algorithm was trained separately on two different datasets (soft tissue lesions and calcifications), and later on, it was combined. This was clearly seen in the study by Lotter et al., where they presented a multi-scale convolutional neural network scanning window scheme with a lesion-specific curriculum learning strategy that showed promising results [33]. They demonstrated that their approach effectively handles the “needle in a haystack” nature of full-image mammogram classification, achieving 0.92 area under the receiver operating characteristic (AUROC) curve on the digital database for screening mammography dataset. On the other hand, there have been algorithms that use one-image-level supervision and are trained by weak supervision. Eun-Kyung et al. used this approach and found a sensitivity of 76.1% and a specificity of 88.5% for both screening and diagnostic mammograms [34].

Algorithms operating at the pixel or patch level often necessitate outlining the location of malignant lesions, with one common method being transfer learning. Transfer learning involves training the entire network but initializing the weights from pre-trained weights on other datasets for the new system, as demonstrated in the study by Samala et al. [35]. They applied knowledge gleaned from non-medical images to medical diagnostic tasks through supervised training, enhancing the algorithm's capabilities by concurrently learning other tasks. The study concluded that multi-task transfer learning could be an effective approach for training deep convolutional neural networks in medical imaging applications, especially when training samples from a single modality are limited. Additionally, Becker et al. utilized multi-purpose image analysis software originally intended for industrial use, training the algorithm to detect cancer in mammograms using two datasets [36]. Their results revealed that the AUROC curve of the trained neural network was 0.81, comparable to 0.79 in the test cases (p = 0.63). While one radiologist exhibited nearly equal performance (0.83, p = 0.17), two radiologists showed significantly better performance (0.91 and 0.94, p < 0.016). Furthermore, the neural network's performance (0.82) did not significantly differ from human performance (0.77-0.87, p > 0.016). However, radiologists consistently demonstrated lower sensitivity and higher specificity compared to the neural network, indicating the network's ability to detect cancer in mammograms with accuracy comparable to radiologists, especially in scenarios of low breast cancer prevalence.

More recent algorithms have been proposed where analysis is done across the two breasts and the inclusion of prior exams. Kooi and Karssemeijer employed a simple linear mapping technique that takes the location of a mass and maps it to either the contralateral or to a prior mammogram [37]. As mentioned above, even though the use of temporal comparison is emphasized in clinical practice, it has been shown in a few studies to not change patient care [13-16]. Thus, further work is needed to determine if having an algorithm that performs temporal comparison will have clinical significance. It may very well be that future algorithms are able to detect some biomarkers that the human eye is not able to detect from prior images. 

Clinical implementation of deep learning

Presently, multiple companies are offering FDA-approved commercial AI applications, with some nearing the final stages of market readiness. Lotter et al. devised a deep learning approach that underwent progressive training stages while retaining interpretability based on localization [38]. In their reader study, the system surpassed five out of five breast radiologists in performance. Additionally, Rodríguez-Ruiz et al. compared the breast cancer detection capabilities of radiologists reading mammograms unaided versus with AI assistance [39]. Their findings revealed that radiologists enhanced their cancer detection rates when supported by an AI system during mammography readings without necessitating extra reading time. Both sensitivity and specificity showed improvements with AI support, while the overall reading time for the entire dataset remained similar. Consequently, AI algorithms hold the potential for parallel use during readings, enabling radiologists to allocate more time to complex cases.

The ideal algorithm

In light of the low prevalence of breast cancer in screening populations, we advocate for an optimal AI algorithm capable of operating with high sensitivity, thereby yielding a high negative predictive value. This approach enables screening radiologists to allocate more time to suspicious cases, potentially mitigating the shortages faced by some countries in radiologist staffing. The primary aim of the AI algorithm is to enhance patient care by improving real-time breast cancer detection and addressing cases currently overlooked by radiologists. Notably, Rodríguez-Ruiz et al. investigated the feasibility of automatically identifying normal mammography exams using AI to reduce the workload of breast cancer screening readings. They demonstrated the potential for a significant reduction in workload through automatic exam preselection using AI. However, a significant drawback of preselection strategies arises when radiologists interpret cases, as they are aware that the AI flagged the case due to suspicions. While incorporating AI into breast cancer screening holds promise for improving early detection and alleviating radiologist shortages, challenges such as potential bias and its impact on radiologists' performance must be addressed. Future research and collaboration are essential to strike the right balance between AI assistance and human expertise in this critical medical domain [40].

Use of AI in the management and treatment of breast cancer

Surgical Management

The wide range of applications of AI in breast surgery offers significant potential, prompting surgeons to remain updated on advancements to enhance their clinical methods, thereby enhancing patient results. Artificial intelligence tools like chatbots have shown convincing results in pre-, intra-, and postoperative breast reconstruction. The integration of AI in these operations could play a crucial role in the comprehensive approach to treating breast cancer, focusing on restoring a patient's body image and improving quality of life post-surgeries like mastectomy and lumpectomy. Artificial intelligence's potential in breast cancer screening can significantly reduce both error rates and physician workload. Artificial intelligence assists in evaluating factors like breast volume and shape, aids in reconstructive surgery planning, and can simulate cosmetic outcomes preoperatively, offering insights into potential results. While intraoperative AI assistance has not been extensively explored in breast reconstruction, AI aids postoperative care through early complication detection, predicting complications, and assessing postoperative pain. In a retrospective study conducted by Zhu et al., the multifactor AI model developed by their team showed remarkable effectiveness in identifying axillary lymph node metastasis, marking a breakthrough in surgical research [41]. Notably, the study introduced an AI-supported surgical approach that significantly enhanced the model's accuracy compared to the conventional sentinel lymph node biopsy method alone. This suggests that employing AI in surgical decision-making regarding the axilla could be a valuable and efficient strategy for breast cancer cases post-chemotherapy [42]. The present focus must be on the amalgamation of AI in preoperative planning, analyzing patient-specific data for surgical approaches, guiding real-time imaging for precise tumor resections, and monitoring postoperative stages for early complication detection and improved standards of care [43].

Radiation Oncology

Artificial intelligence can support radiation oncologists by aiding in the preparation of radiation therapy delivery, leveraging data from various sources like images, treatment algorithms, and dose-volume parameters, thereby enhancing this crucial aspect of breast cancer treatment. As mentioned above, AI algorithms aid in interpreting mammograms, thereby offering improved accuracy in identifying lesions. Furthermore, AI can also help in predicting treatment responses, assisting clinicians in devising personalized therapy plans based on individual patient characteristics and tumor behavior. Artificial intelligence has proven its worth by automating the outlining of organs crucial for treatment and defining target volumes. Additionally, it aids in automatically planning the distribution of radiation doses, resulting in time savings, enhanced consistency, and better parameters for dose volumes. The next phase involves integrating dose and outcome data to refine dose distributions. This aims to achieve optimal coverage of high-risk areas while limiting doses to low-risk areas by tailoring radiation doses based on spatial adjustments. This approach allows for the creation of a flexible range of volumes and doses, minimizing radiation exposure to organs considered at risk. To accomplish this, various patient, tumor, and treatment factors need to be combined with databases containing dose distributions and outcomes [44]. A study conducted by Velzen et al. highlighted the utility of an AI-based radiation dose quantification model that automatically segments cardiac structures, providing accurate localized dose estimations and offering significant potential to prevent radiation-induced cardiac damage in breast cancer patients. Computed tomography scans and radiation data from over 5,000 patients were analyzed to study the impact of radiation on heart disease in breast cancer patients. Higher radiation doses to cardiac structures are correlated with increased heart disease risk, especially in patients with existing coronary artery calcification, though no direct interaction between calcification and radiation dose was found [45]. Therefore, AI has the potential to supplement the prevailing use of CT in data management and analysis.

Medical Oncology

A study by Dodington et al. utilized AI to analyze nuclear features in breast cancer biopsies to predict response to neoadjuvant chemotherapy. Generally, in breast cancer treatment, neoadjuvant chemotherapy is administered before surgery to shrink tumors. Predicting a patient's response to this treatment is crucial, as it helps doctors personalize therapy and improve outcomes. The findings highlighted specific nuclear characteristics that, when analyzed through digital pathology, significantly enhanced the prediction of pathological response to neoadjuvant chemotherapy, potentially aiding in treatment planning for the same [46]. In a study conducted by Wan et al., a novel AI-driven model involving genes successfully predicted both the effectiveness of chemotherapy and the crucial role of the cdkn2a/magea4 pathway [45]. It explored the prognosis of how changes in cdkn2a might affect resistance to chemotherapy, particularly in relation to the MAGE-A family. Using an AI-constructed pan-cancer risk model, it aimed to understand how these genes interact across different cancers, potentially shedding light on the mechanisms behind treatment resistance [47]. Hence, the integration of AI-driven analyses of nuclear features and genetic pathways presents promising avenues for better treatment strategies in breast cancer patients undergoing chemotherapy.

Current limitations and the future of mammography and AI

While digital mammography has been advantageous in the realm of breast cancer screening, there are limitations to its use. These include overdiagnosis, overtreatment, and false-positive rates with associated psychological impact and unnecessary costs and biopsies [48-51]. Additionally, difficult interpretations of mammograms are subjective and based on the experience of the radiologist. Thus, it is difficult at times to interpret and prone to error depending on the heterogeneous breast cancer presentation and density. Given these limitations of digital mammography, we hope that AI algorithms can be used to minimize the variables prospectively. 

Another potential area of interest for AI development in breast cancer detection that needs further work is mammographic risk assessment metrics. The most common ones that are used are based on Wolfe, Boyd, Tabár, and the Breast Imaging Reporting and Data System (BI-RADS) [52-54]. Boyd’s measures the percentage area of dense breast tissue. Whereas Wolfe, BI-RADS, and Tabár all include patterns and texture information. To our knowledge, there is currently minimal literature on this area of AI development, which could potentially serve as a beneficial aid to radiologists in breast cancer screening. Chunyan et al. demonstrated that the AI system can effectively assist mid-level radiologists in reducing unnecessary follow-ups of mammographically indeterminate breast lesions and reducing the benign biopsy rate without missing highly malignant tumors in BI-RADS 0 scans [55].

A summary of studies on AI applications in breast cancer screening, diagnosis, and treatment is presented in Table 1.

**Table 1:** Summary of studies on artificial intelligence (AI) applications in breast cancer screening, diagnosis, and treatment

Studies	Title	Year
Ghoncheh et al. [1]	Incidence and mortality and epidemiology of breast cancer in the world	2016
DeSantis et al. [2]	Breast cancer statistics, 2017, racial disparity in mortality by state	2017
Independent UK Panel on Breast Cancer Screening [3]	The benefits and harms of breast cancer screening: an independent review	2012
Tabár et al. [5]	Insights from the breast cancer screening trials: how screening affects the natural history of breast cancer and implications	2014
Zeeshan et al. [6]	Diagnostic accuracy of digital mammography in the detection of breast cancer	2018
Healy et al. [7]	Consensus review of discordant imaging findings after the introduction of digital screening mammography	2020
Posso et al. [8]	Effectiveness and cost-effectiveness of double reading in digital mammography screening: a systematic review and meta-analysis	2017
Séradour et al. [10]	Comparison of direct digital mammography, computed radiography, and film-screen in the French national breast cancer screening	2014
Pisano et al. [11]	Digital Mammographic Imaging Screening Trial (DMIST) investigators group	2005
Sumkin et al. [13]	Optimal reference mammography: a comparison of mammograms obtained 1 and 2 years before the present examination	2003
Thurfjell et al. [14]	Effect on sensitivity and specificity of mammography screening with or without comparison of old mammograms	2000
Roelofs et al. [15]	Importance of comparison of current and prior mammograms in breast cancer screening	2007
Varela et al. [16]	Use of prior mammograms in the classification of benign and malignant masses	2005
Destounis et al. [17]	Initial experience with combination digital breast tomosynthesis plus full field digital mammography or full field digital mammography alone in the screening environment	2014
Durand et al. [18]	Tomosynthesis-detected architectural distortion: management algorithm with radiologic-pathologic correlation	2016
Zackrisson et al. [19]	One-view breast tomosynthesis versus two-view mammography in the Malmö Breast Tomosynthesis Screening Trial	2018
Hofvind et al. [20]	Digital breast tomosynthesis and synthetic 2D mammography versus digital mammography: evaluation in a population-based screening program	2018
Skaane et al. [21]	Comparison of digital mammography alone and digital mammography plus tomosynthesis in a population-based screening program	2013
Gilbert et al. [22]	Accuracy of digital breast tomosynthesis for depicting breast cancer subgroups in a UK retrospective reading study (TOMMY Trial)	2015
Ciatto et al. [23]	Integration of 3D digital mammography with tomosynthesis for population breast-cancer screening (STORM): a prospective comparison study	2013
Bernardi et al. [24]	Breast cancer screening with tomosynthesis (3D mammography) with acquired or synthetic 2D mammography compared with 2D mammography alone (STORM- 2): a population-based prospective study	2016
Lång et al. [25]	Performance of one-view breast tomosynthesis as a stand-alone breast cancer screening modality: results from the Malmö Breast Tomosynthesis Screening Trial, a population-based study	2016
Good et al. [26]	Digital breast tomosynthesis: a pilot observer study	2008
Gur et al. [27]	Digital breast tomosynthesis: observer performance study	2009
Conant et al. [28]	Improving accuracy and efficiency with concurrent use of artificial intelligence for digital breast tomosynthesis	2019
Benedikt et al. [29]	Concurrent computer-aided detection improves reading time of digital breast tomosynthesis and maintains interpretation performance in a multireader multicase study	2018
Lehman et al. [30]	Diagnostic accuracy of digital screening mammography with and without computer-aided detection	2015
Fenton et al. [31]	Effectiveness of computer-aided detection in community mammography practice	2011
Sechopoulos et al. [32]	Artificial intelligence for breast cancer detection in mammography	2021
Lotter W et al [33]	A multi-scale CNN and curriculum learning strategy for mammogram classification	2017
Kim et al. [34]	Applying data-driven Imaging biomarker in mammography for breast cancer screening: preliminary study	2018
Samala et al. [35]	Multi-task transfer learning deep convolutional neural network: application to computer-aided diagnosis of breast cancer on mammograms	2017
Becker et al. [36]	Deep learning in mammography: diagnostic accuracy of a multipurpose image analysis software in the detection of breast cancer	2017
Kooi and Karssemeijer [37]	Classifying symmetrical differences and temporal change for the detection of malignant masses in mammography using deep neural networks	2017
Lotter et al. [38]	Robust breast cancer detection in mammography and digital breast tomosynthesis using an annotation-efficient deep learning approach	2021
Rodriguez-Ruiz et al. [39]	Stand-alone artificial intelligence for breast cancer detection in mammography: comparison with 101 radiologists	2019
Rodriguez-Ruiz et al. [40]	Can we reduce the workload of mammographic screening by automatic identification of normal exams with artificial intelligence? A feasibility study	2019
Seth et al. [41]	Use of artificial intelligence in the advancement of breast surgery and implications for breast reconstruction: a narrative review	2023
Zhu et al. [42]	Multifactor artificial intelligence model assists axillary lymph node surgery in breast cancer after neoadjuvant chemotherapy: multicenter retrospective cohort study	2023
Akalın Ç [43]	Advances in artificial intelligence and the potential impact on oncoplastic breast surgery	2023
Poortmans et al. [44]	Winter is over: the use of artificial intelligence to individualise radiation therapy for breast cancer	2020
van Velzen et al. [45]	AI-based quantification of planned radiation therapy dose to cardiac structures and coronary arteries in patients with breast cancer	2022
Dodington et al. [46]	Analysis of tumor nuclear features using artificial intelligence to predict response to neoadjuvant chemotherapy in high-risk breast cancer patients	2021
Wan et al. [47]	CDKN2A was a cuproptosis-related gene in regulating chemotherapy resistance by the MAGE- a family in breast cancer: based on artificial intelligence (AI)-constructed pan-cancer risk model	2023
Jørgensen and Gøtzsche [48]	Overdiagnosis in publicly organised mammography screening programmes: systematic review of incidence trends	2009
Bond [49]	Refining Lewin’s formula: A general model for explaining situational influence on individual social behavior	2013
Tosteson et al. [50]	Consequences of false-positive screening mammograms	2014
Pharoah et al. [51]	Cost effectiveness of the NHS breast screening programme: life table model	2013
Wolfe [52]	Risk for breast cancer development determined by mammographic parenchymal pattern	1976
Boyd et al. [53]	Quantitative classification of mammographic densities and breast cancer risk: results from the Canadian National Breast Screening Study	1995
Jong [54]	Breast cancer: the art and science of early detection with mammography	2006
Yi et al. [55]	The added value of an artificial intelligence system in assisting radiologists on indeterminate BI-RADS 0 mammograms	2022

Despite the ongoing research into integrating AI into breast cancer screening, medico-legal and ethical considerations remain paramount. Presently, when a pre-selection threshold is established (without the radiologist's involvement), the performance of AI and a radiologist is roughly comparable. However, this threshold may result in the AI missing certain cancers.

To clarify, both the radiologist and the AI system may overlook an equal number of cases in a breast cancer screening population, albeit different ones. Whether this poses a significant problem hinges on the type of breast cancer detected and missed by both parties. Further assessment is imperative to ascertain the long-term implications. Moreover, addressing the question of responsibility from a legal standpoint necessitates the implementation of quality control protocols for AI algorithms and regular performance audits conducted by radiologists [56].

## Conclusions

Initially, CADs were introduced; however, they did not show any long-term benefit in clinical practice. Over time, convolutional neural network algorithms have significantly improved, resulting in an increase in sensitivity and specificity in breast cancer detection. Given the limitations in the literature currently regarding all studies being retrospective, it has not been fully clear whether this system can be beneficial to breast radiologists in a real-time setting. This can only be evaluated by performing a prospective study and seeing in what situations the system works optimally. To truly gauge the system's effectiveness in real-time clinical practice, prospective studies are necessary to address current limitations stemming from retrospective data. These studies will help identify the optimal conditions for AI-assisted breast cancer screening and pave the way for a more comprehensive understanding of the impact of deep learning on clinical practice.
